# Clinic follow-up of orthopaedic trauma patients during and after the post-surgical global period: a retrospective cohort study

**DOI:** 10.1186/s12891-023-06218-y

**Published:** 2023-02-13

**Authors:** Abhiram R. Bhashyam, Sravya T. Challa, Hannah Thomas, Edward K. Rodriguez, Michael J. Weaver

**Affiliations:** 1 Department of Orthopaedic Surgery, Massachusetts General Hospital, Harvard Orthopaedic Trauma Initiative, Harvard Medical School, 55 Fruit St, Boston, MA 02114 USA; 2 Department of Orthopaedic Surgery, Massachusetts General Hospital, Boston, MA USA; 3 Harvard Medical School, Boston, MA USA; 4 Department of Orthopaedic Surgery, Beth Israel Deaconess Medical Center, Harvard Orthopaedic Trauma Initiative, Harvard Medical School, Boston, MA USA; 5 Department of Orthopaedic Surgery, Brigham and Women’s Hospital, Harvard Orthopaedic Trauma Initiative, Harvard Medical School, Boston, MA USA

**Keywords:** Financial burden, Orthopaedic trauma, Follow-up, Fracture, Insurance

## Abstract

**Background:**

Insurance status is important as medical expenses may decrease the likelihood of follow-up after musculoskeletal trauma, especially for low-income populations. However, it is unknown what insurance factors are associated with follow-up care. In this study, we assessed the association between insurance plan benefits, the end of the post-surgical global period, and follow-up after musculoskeletal injury.

**Methods:**

This is a retrospective cohort study of 394 patients with isolated extremity fractures who were treated at three level-I trauma centers over four months in 2018. Paired t-tests were utilized to assess the likelihood of follow-up in relation to the 90-day post-surgical global period. Regression analysis was used to assess factors associated with the likelihood of follow-up. Supervised machine learning algorithms were used to develop predictive models of follow-up after the post-surgical global period.

**Results:**

Our final analysis included 328 patients. Likelihood of follow-up did not significantly change while within the post-surgical global period. When comparing follow-up within and outside of the post-surgical global period, there was a 20.1% decrease in follow-up between the 6-weeks and 6-month time points (68.3% versus 48.2%, respectively; *p* < 0.0001). Medicaid insurance compared to Medicare (OR 0.27, 95% confidence interval (CI) = [0.09, 0.84], *p *= 0.02) was a predictor of decreased likelihood of follow-up at 6-months post-operatively.

**Conclusions:**

Our study demonstrates a statistically significant decrease in follow-up for orthopaedic trauma patients after the post-surgical global period, particularly for patients with Medicaid or Private insurance.

## Background

In the United States, large out-of-pocket expenses associated with medical care result in decreased healthcare equity and access to necessary services, especially for low-income patients [1, 2]. In Massachusetts, recent health reforms led to an increased number of insured individuals [3, 4]. However, even among insured patients, the amount of financial burden caused by medical expenditures varies considerably [5]. When considering treatment adherence after unexpected musculoskeletal trauma, financial burden is especially important as medical expenses may prevent low-income populations from accessing follow-up services. Despite this link between insurance type and financial burden, it is still unclear if insurance is associated with post-injury follow-up care.

Orthopedic trauma presents unique financial burdens to patients, as musculoskeletal injury is unexpected and requires prompt care, often without adequate time to accommodate personal convenience and financial preparedness. Non-ambulatory patients often do not have a choice in provider, and are taken by emergency medical services to the nearest trauma center. As a result, many patients are logistically unable to ensure they are treated by a provider within their insurance network. Furthermore, orthopaedic trauma can lead to non-working status and physical disability, which can impact a patient’s financial health in both the short and long term [6, 7]. A recent study found that despite a high rate of insurance, these patients experienced a high rate of worry and financial distress secondary to injuries, and metrics of financial distress were independently associated with insurance type [5]. Recovery from orthopaedic trauma often requires considerable rehabilitation prior to being able to return to work, and thus can be associated with long-lasting or even permanent functional limitations [8]. Follow-up care is important to monitor post-operative recovery and appropriately adapt treatment plans [9, 10]. Yet, it is known that follow up after orthopaedic trauma care is poor [11, 12].

Recent studies have shown that patients with non-private insurance are less likely to attend follow-up appointments [11]. Therefore, in this study, we assessed the association between insurance plan benefits and coverage with follow-up after musculoskeletal injury. We hypothesized that decreased clinic follow-up after treatment for musculoskeletal trauma would be associated with the end of the 90-day post-surgical global period and some types of insurance.

## Methods

This study was approved by our institutional review board and reported using STROBE guidelines. A retrospective cohort study of 394 patients who were treated at three American College of Surgeons level-1 trauma centers within the same geographic region over four months in 2018 was performed. Patients aged 18 years or older with an isolated extremity fracture treated as an inpatient following admission from the emergency department were included. This cohort of patients was expected to have at least 6-month follow-up duration. Exclusion criteria were multiple injuries, admission to non-orthopedic services, non-surgical fracture management, outpatient surgery, elective admission, and insurance plans that restricted follow-up at our institution.

For the patients in this cohort, the global period was 90 days (i.e. 3 months). Our outcome variables were 2-week follow-up, 6-week follow-up, 3-month follow-up, 6-month follow-up, and 1-year follow-up after surgery. Detailed demographic and clinical data were identified for each patient using our institutions’ Research Patient Data Registry and the electronic medical record. The following information was extracted for each patient: age, sex, race, level of education, comorbidities, fracture type, surgery type, hospital duration, complications, insurance plan name, and dates of follow up visits. Patient comorbidity was calculated using the Charlson Comorbidity Index (CCI). Fracture type was classified as upper extremity fracture or lower extremity fracture/pelvis/acetabulum. Surgery type was coded as external fixation or external fixation followed by open reduction internal fixation (ORIF), ORIF with a plate/screw construct, ORIF with an intramedullary nail, arthroplasty or other.

Insurance plan benefits and coverage were obtained from publicly available summary of benefits and coverage documents for each health plan. We recorded plan name, association with a health savings account, annual deductible, out-of-pocket maximum, co-pay for an emergency visit, co-pay for a specialist clinic visit, imaging coverage, durable medical equipment coverage, and physical and occupational therapy coverage. For statistical analysis, primary health insurance was divided into 3 categories (Private, Medicare, and Medicaid).

### Statistical analysis

Paired t-tests were utilized to assess the likelihood of follow-up in relation to the post-surgical global period. First, we assessed differences in follow-up within the global period (2-week compared to 6-week follow-up). Next, we assessed the likelihood of follow-up within the global period (6-week follow-up) versus follow-up clearly outside of the global period (6-month follow-up). We also assessed differences in follow-up at time points before and just after the post-surgical global period (6-week follow-up versus 3-month follow-up). Finally, we assessed differences in follow-up when outside of the post-surgical global period (3-month follow-up versus 6-month follow-up; 6-month follow-up versus 1-year follow-up). We accounted for multiple testing by utilizing ANOVA, as well as Bonferroni correction. Therefore, significance was set at *p* = 0.01 (*p* = 0.05 divided by 5 comparisons = 0.01) for this analysis.

We used regression analysis with generalized linear models to determine factors associated with the likelihood of follow-up (within the 90-day global period and after the 90-day global period up to 6 months since this was the expected follow-up duration for this cohort). Six pre-selected parameters were utilized a priori in the models: age at surgery, sex, insurance-type, age-adjusted CCI, deductible, and level of education. Six parameters were selected based on the general principle of 50 events per predictor. Significance was set at *p* < 0.05. Stata software, version14 (StataCorp) was used for all statistical analyses.

### Developing predictive models

Supervised machine learning algorithms for follow-up after the post-surgical global period (6-month follow-up) were developed using an 80:20 split for training (80%) and testing (20%) sets following TRIPOD guidelines [13]. Using all available data, the following algorithms were utilized: 1. Neural network, 2. Random forest, 3. Gradient boosting machine, and 4. Naïve Bayes classifier. Model performance for training and testing data sets was assessed using discrimination (accuracy, F1 score and area under curve or AUC), calibration (plot, slope and intercept) and overall performance (Brier score). Alteryx Designer software, version 2021.1 (Alteryx) was used for all predictive modeling using machine learning.

## Results

Three hundred and twenty-eight patients were included in the final analysis (Table 1). The majority of the patients in our study were male (64.6%) and White (85.7%), had a High School or College degree (62.8%) and sustained lower extremity fractures (96.4%). The average Charlson Comorbidity Index (CCI) was 3.5 [14].

**Table 1:** Demographic Information. *N* = number of patients, % = percentage of patients, *SD* = standard deviation

	**Patients who completed follow-up**		**Patients who were lost to follow-up**	
**Demographics**	N	% (SD)	N	% (SD)
**Gender**				
Male	93	34.8	23	37.7
Female	174	65.2	38	62.3
**Race**				
White	226	84.6	55	90.2
African American	10	3.8	3	4.9
Asian	6	2.3	0	0
American Indian/Alaskan Native	1	0.4	0	0
Native Hawaiian or other Pacific Islander	1	0.4	0	0
Other/Unknown	23	8.6	3	4.9
**Education**				
Some education, no diploma or GED	15	5.6	5	8.2
High school graduate (diploma or equivalent GED)	78	29.2	23	37.7
Some college, no degree	26	9.7	10	16.4
Associates degree	0	0	1	1.6
Bachelors degree	60	22.5	8	13.1
Masters degree	21	7.9	4	6.6
Doctorate or professional degree	2	0.8	0	0
Other	65	24.3	10	16.4
**Average Charlton Comorbidity Index (CCI)**	3.3	(2.6)	4.3	(2.4)
**Insurance**				
Medicare	123	46.1	36	59
Masshealth/Ancillaries	21	7.8	1	1.6
Low deductible private health plan	93	34.8	21	34.4
High deductible private health plan	30	11.2	3	4.9
Uninsured				
**Mechanism of Injury**				
Ground Level Fall	196	73.4	47	78.3
High energy fall	25	9.4	6	10
MVC	10	3.8	1	1.7
Motorcycle accident	8	3	0	0
Sports-related	2	0.8	0	0
Pedestrian struck	3	1.1	1	1.7
Bicycle accident	5	1.9	0	0
Direct blow	0	0	1	1.7
Other	18	6.7	4	6.7

### Likelihood of follow up by time

**Fig. 1:**
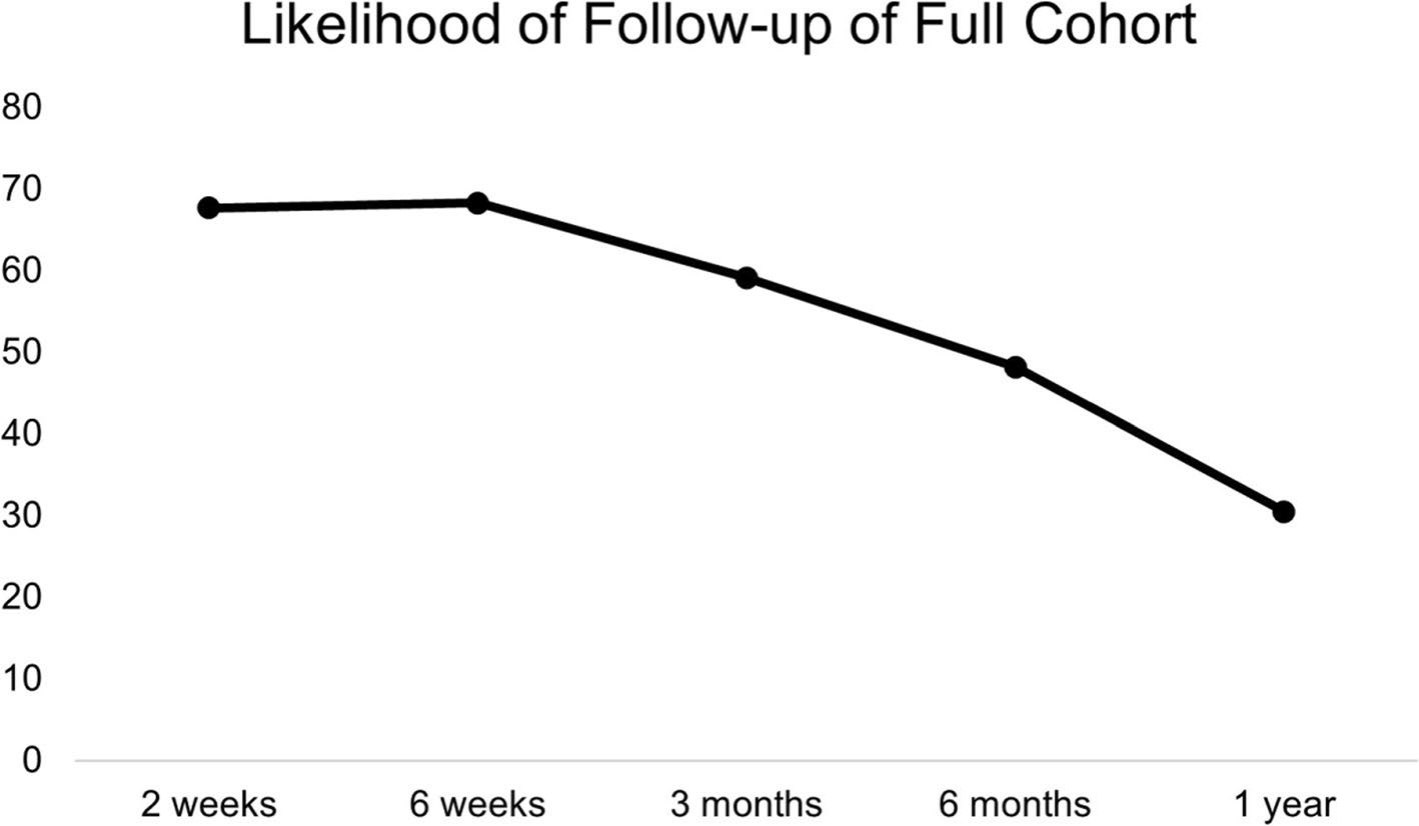
Likelihood of follow up of the full cohort by time

In our cohort, likelihood of follow up did not significantly change between 2 and 6 weeks while within the post-surgical global period. Follow-up progressively decreased between 6 weeks and one year postoperatively (Fig. 1). When comparing follow-up within and outside of the post-surgical global period, there was a 20.1% decrease in follow up between the 6-weeks and 6-month time points (68.3% versus 48.2%, respectively; *p* < 0.0001). Similarly, there was a 9.1% decrease in follow-up between 6-weeks and 3-months (68.3% versus 59.1%, respectively; *p* = 0.004). Follow-up continued to decrease significantly when outside of the global period as there was a 11% decrease in follow up between 3 months (59.2%) and 6 months (48.2%, *p* < 0.001). Follow-up continued to significantly decrease between 6 months (48.2%) and 1 year (30.5%) postoperatively (*p* < 0.0001).

### Predictors of follow-up at 2 weeks, 6 weeks, 3 months and 6 months

Using generalized linear models, age was the only significant predictor for follow-up within the global period at 2 weeks, 6 weeks or 3 months postoperatively (OR 0.96, 95% confidence interval = [0.95, 0.99], *p* = 0.004). After the global period, Medicaid insurance (compared to Medicare; OR 0.27, 95% confidence interval = [0.09, 0.84], *p* = 0.02) was a predictor of decreased likelihood of follow-up at 6-months post-operatively. Injury/fracture type was not related to length of follow-up.

### Predictive models of follow-up at 6 months

**Fig. 2:**
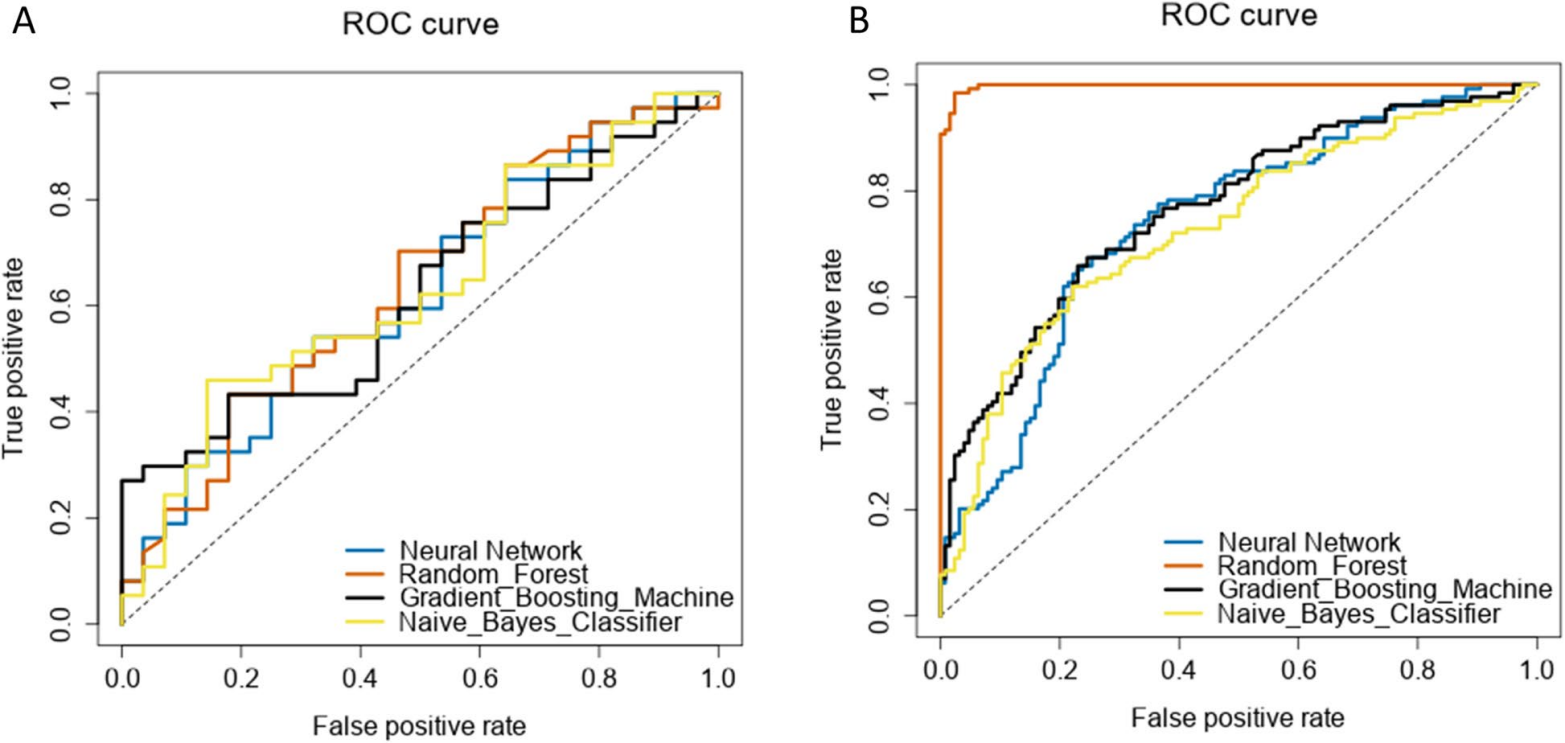
ROC curve for Training (**A**) and Testing (**B**) data sets for the predictive models indicative of discrimination, referring to the model’s ability to assess loss of follow up

**Fig. 3:**
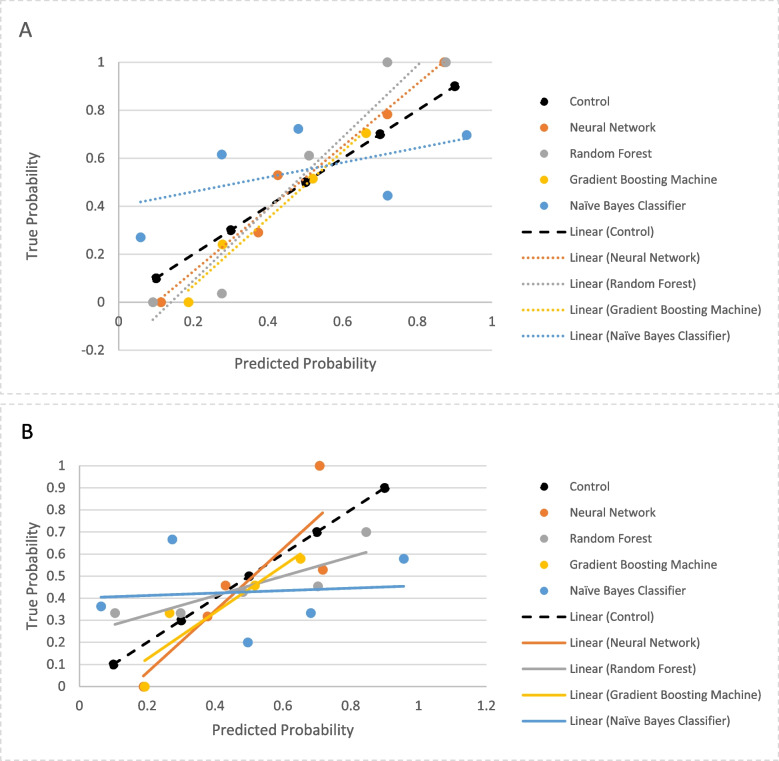
Calibration curve for predictive models; **A** Testing dataset, **B** Training dataset. Calibration is a measure of how well the model's predicted probabilities compare to observed probabilities in the study population. Calibration slope measures the difference between predictor effects for the model in training and testing sets; a calibration slope of 1 indicates that the predictor effects for the model are equivalent in both sets

For the training data set, the AUC for the models ranged from 0.63 for the Gradient Boosting Machine model to 0.99 for the Random Forest model (Fig. 2A). The Brier score ranged from 0.26 for the Naïve Bayes Classifier to 0.06 for the Random Forest model (Table 2). Calibration curves for the training models are presented in Fig. 3A.

**Table 2:** Performance of predictive models on the training and testing dataset

	**Gradient Boosting Machine**	**Naïve Bayes Classifier**	**Neural Network**	**Random Forest**
*Training*				
Brier score	0.20	0.26	0.22	0.06
F1 score	0.70	0.66	0.71	0.98
Sensitivity	0.77	0.59	0.34	0.97
Specificity	0.63	0.73	0.90	0.98
Positive Predictive Value	0.67	0.68	0.77	0.98
Negative Predictive Value	0.74	0.64	0.58	0.97
AUC	0.628	0.729	0.741	0.998
*Testing*				
Brier score	0.24	0.33	0.24	0.24
F1 score	0.56	0.62	0.67	0.65
Sensitivity	0.57	0.39	0.39	0.54
Specificity	0.51	0.65	0.73	0.65
Positive Predictive Value	0.47	0.46	0.52	0.54
Negative Predictive Value	0.61	0.59	0.61	0.65
AUC	0.626	0.632	0.625	0.639

**Fig. 4:**
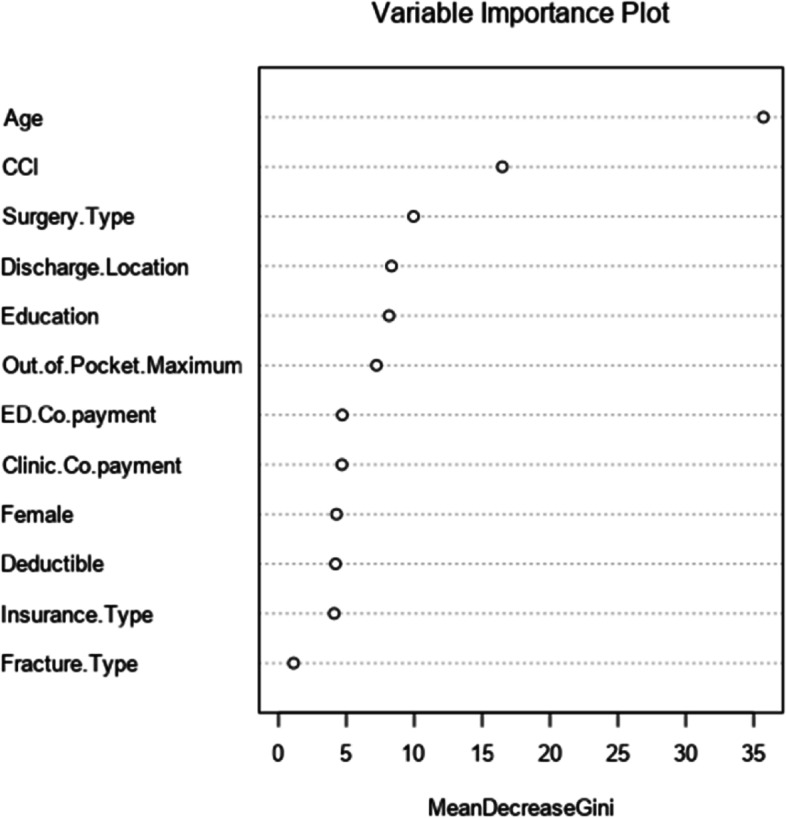
Variable importance plot for follow-up at 6-months based on the Random Forest predictive model

For the testing dataset, the AUC for the models ranged from 0.63 for the Neural Network and Gradient Boosting Machine models to 0.64 for the Random Forest model (Fig. 2B). The Brier score ranged from 0.33 for the Naïve Bayes Classifier to 0.24 for the Neural Network, Random Forest and Gradient Boosting Machine models (Table 2). Calibration curves for the models are presented in Fig. 3B. Based on the training and testing datasets, the Random Forest model was the best predictive model for follow up and had the best performance across discrimination, calibration and overall performance. In this model, the insurance factor most strongly correlated with follow-up at 6 months was the out-of-pocket maximum, although this effect size was smaller than other demographic- and treatment-related factors (Fig. 4).

## Discussion

In this study, we demonstrated that the post-surgical global period is a critical timepoint influencing follow-up rates in orthopedic trauma patients, and follow-up at 6 months was lowest for patients on Medicaid. As follow-up care after surgery is critical to monitor recovery and appropriately adapt the course of treatment [9, 10], knowledge of factors that decrease follow-up is important so that clinicians can identify at-risk patients and insurers might reshape plan details to increase equitable access to follow-up care. Our results suggest that decreased long-term follow-up in this patient population is likely influenced by health insurance plan factors, such as higher out-of-pocket maximums and lack of post-surgical coverage beyond 90-days. Future research is needed into whether targeted changes to lengthen the global post-surgical coverage period, patient education, or telemedicine visits can improve access to necessary follow-up care.

Long-term follow up for orthopedic trauma patients is a known challenge. One recent study looking at likelihood of follow up in orthopedic trauma patients found that the one-year rate of follow up was 29%. In their study, risk factors for decreased follow-up were homelessness, drug or tobacco use and lack of commercial insurance [12]. Building on this study, our data suggest that follow up rate is also significantly lower after the 90-day global period, even for patients who are expected to follow-up for 6 months. This is particularly significant for the clinical care of periarticular injuries since weight-bearing often does not begin for 6–12 weeks from surgery and radiographic union of fractures occurs between 3–6 months. For this patient population, follow-up beyond the 90-day global period is clinically relevant and it will be especially important to develop mechanisms to bridge the gap in insurance coverage.

Recent studies have shown that the personal financial burden associated with medical care is substantial. A national study by the U.S. Department of Health and Human Services and the U.S. Centers for Disease Control and Prevention (CDC) found that over one fourth of American families reported having experienced financial burden because of medical care and 1 in 5 Americans had a family member who had trouble paying medical bills. Though data on financial burden among patients with musculoskeletal trauma are limited, an earlier study found that despite a high rate of insurance, these patients experienced a high rate of worry and financial distress secondary to injuries. In both studies, patients with Medicaid plans were at higher risk for greater levels of financial burden and decreased likelihood of follow-up [5]. Medicaid insurance may be an indicator of patients who may be predisposed to financial distress or underinsured status [15]. For these patients, increased additive costs outside of the global period and higher out of pocket maximums may result in increased financial stress leading to decreased follow-up. There may also be patient characteristics within Medicaid or underinsured populations that predispose to decreased likelihood of follow-up. In reciprocal fashion, there may also be unconscious bias among providers in the treatment of these patients, especially in follow-up.

In addition to reshaping insurance plan details to facilitate longer covered access to post-surgical care, patient education is likely key to increasing health equity after musculoskeletal trauma. By ensuring that a patient knows the benefits of follow-up and has the necessary tools to be able to follow-up, clinicians may help to improve access. Patients may benefit from education surrounding the expected progression of recovery and signs of complication, so that they can prioritize follow-up when it is most needed. While electronic patient portals are useful tools to communicate with and educate patients, low-income groups and minorities still have the lowest usage of these platforms [16, 17]. Clinicians must consider inequities in usage of online patient portals when using these tools for patient care. Another possible mechanism to improve follow-up is increasing the availability of virtual visits for routine care, which would decrease the amount of time and money that patients spend commuting to follow-up visits, which may have contributed to loss of follow up, especially in older age patients [18, 19]. Further research is needed into how to most effectively increase follow-up equity using technology, patient education, and improved benefit design. Compared to other countries where 1-year follow-up is 75% or greater, our study also supports previous findings that challenges with follow-up in the United States are likely driven by system and patient-related issues [20, 21]. Some form of structural change will likely be required to fully address this problem because surgeons are limited in their ability of fully ameliorate the costs of follow-up care. Even if a surgeon waives their professional fee, there may still be facility fees or costs associated with radiology and durable medical goods that are uncontrollable by the individual physician.

In addition to telemedicine, predictive machine learning models can highlight salient risk factors and help us consistently identify patients most at risk for loss to follow-up or inadequate care. While typical predictors like age, medical comorbidities, and surgery type were identified as factors associated with follow-up, our models also demonstrate the importance of insurance-related factors. While most patients were insured in this study, we found that higher out-of-pocket maximums and co-payments were also associated with decreased follow-up. This finding suggests that under-insurance continues to be a significant concern for this patient population [5]. Increased provision of healthcare insurance by itself is unlikely to improve the long-term quality of care without changes to insurance plan designs or better targeting of care to at-risk populations. For individual providers and healthcare institutions, the use of unbiased predictive models may allow us to target interventions including timing and type of follow up care, and direct care toward the neediest [22].

### Limitations

This study had several limitations. We excluded 66 patients from our analysis because we were unable to determine insurance details for their specific plan. These plans were recorded in patient electronic health record under a general category (ex: Tufts PPO) rather than a specific plan name (ex: Tufts Advantage PPO Saver), which made finding specific data difficult. Additionally, though the injuries and follow-up included in this study occurred in 2018, we used insurance plan details from 2020 due to information availability. Plan details from prior years were not always publicly available, however, there are minimal changes to plan coverage from year to year. Not all patients are routinely followed for a full year following injury and there may have been some bias in the length of recommended follow-up recommended by the treating surgeon, although all patients in this cohort were recommended to follow-up for at least 6 months. Finally, our study included only patients that received care at an emergency department after their injury. It is possible that some injured uninsured/underinsured patients might seek care elsewhere or even choose to go untreated rather than facing the financial burden associated with hospital-based medical care. Thus, our estimates may understate the true impact of insurance coverage on care-seeking behaviors. Further research is needed to characterize patients who experience traumatic injury but choose not to seek hospital-based care.

## Conclusions

Our study demonstrates a significant decrease in follow-up for orthopaedic trauma patients after the post-surgical global period, particularly in patients with Medicaid insurance. Extended follow-up care is often important to outcomes in musculoskeletal trauma as the recovery and rehabilitation following surgery frequently extends well beyond 3 months. This finding suggests that follow-up may be improved for this population with changes to insurance plan design and coverage.

## Data Availability

The datasets generated and/or analysed during the current study are not publicly available due to IRB patient confidentiality concerns but are available from the corresponding author on reasonable request.
